# Major Depressive Disorder Across Development and Course of Illness: A Functional Neuroimaging Meta-Analysis

**DOI:** 10.1192/j.eurpsy.2023.753

**Published:** 2023-07-19

**Authors:** C. Baten, A. M. Klassen, J. H. Shepherd, G. Zamora, E. Pritchard, S. Saravia, Z. Ali, J. Jordan, S. K. Kahlon, G. Maly, M. Duran, S. Santos, A. F. Nimarko, D. W. Hedges, P. Hamilton, I. H. Gotlib, M. D. Sacchet, C. H. Miller

**Affiliations:** 1 Department of Psychology, California State University, Fresno; 2 Department of Psychology, Stanford University, Stanford; 3 Department of Psychology, Brigham Young University, Provo, United States; 4 Department of Biomedical and Clinical Sciences, Linköping University, Linköping, Sweden; 5 Meditation Research Program, Department of Psychiatry, Massachusetts General Hospital, Harvard Medical School, Boston, United States

## Abstract

**Introduction:**

Functional magnetic resonance imaging (fMRI) has been used to identify the neural activity of both youth and adults diagnosed with major depressive disorder (MDD) in comparison to healthy age-matched controls. Previously reported abnormalities in depressed youth appear to mostly align with those found in depressed adults; however, some of the reported aberrant brain activity in youth has not been consistent with what is observed in adults, and to our knowledge there has not yet been a formal, quantitative comparison of these two groups. In addition, it is not known whether these observed differences between youth and adults with depression are attributable to developmental age or length-of-illness.

**Objectives:**

The aim of this study is to elucidate the similarities and differences in patterns of abnormal neural activity between adults and youth diagnosed with MDD and to then determine whether these observed differences are due to either developmental age or length-of-illness.

**Methods:**

We used multilevel kernel density analysis (MKDA) with ensemble thresholding and triple subtraction to separately determine neural abnormalities throughout the whole brain in primary studies of depressed youth and depressed adults and then directly compare the observed abnormalities between each of those age groups. We then conducted further comparisons between multiple subgroups to control for age and length-of-illness and thereby determine the source of the observed differences between youth and adults with depression.

**Results:**

Adults and youth diagnosed with MDD demonstrated reliable, differential patterns of abnormal activation in various brain regions throughout the cerebral cortex that are statistically significant (p < .05; FWE-corrected). In addition, several of these brain regions that exhibited differential patterns of neural activation between the two age groups can be reliably attributed to either developmental age or length-of-illness.

**Conclusions:**

These findings indicate that there are common and disparate patterns of brain activity between youth and adults with MDD, several of which can be reliably attributed to developmental age or length-of-illness. These results expand our understanding of the neural basis of depression across development and course of illness and may be used to inform the development of new, age-specific clinical treatments as well as prevention strategies for this disorder.

**Disclosure of Interest:**

None Declared

